# Isolated testicular metastasis from prostate cancer following three years of hormone therapy and radiotherapy

**DOI:** 10.1016/j.eucr.2025.103080

**Published:** 2025-05-27

**Authors:** Ali Tabibi, Mohammad Sajjad Zabihi, Mahyar Najarian, Mehdi Dadpour

**Affiliations:** Shahid Labbafinejad Medical Center, The Center of Excellence in Urology, Urology and Nephrology Research center, Research Institute of Urology and Nephrology, Shahid Beheshti University of medical Sciences, Tehran, Iran

**Keywords:** Prostate cancer, Metastasis, Testes

## Abstract

This case report presents a 61-year-old male patient diagnosed with high-risk prostate cancer who underwent hormone therapy and radiotherapy. Three years after beginning treatment, rising prostate-specific antigen (PSA) levels led to a PET CT scan, which identified isolated metastases in the left testicle. The patient then underwent a radical orchiectomy. One-year follow up revealed undetectable PSA and no evidence of any metastases. The significance of this case lies in the uncommon occurrence of testicular metastasis from prostate cancer, particularly when presenting in isolation after a prolonged period following hormone therapy and radiotherapy.

## 1 Introduction

Prostate cancer is one of the most prevalent cancers among men globally, often presenting as localized prostate adenocarcinoma with a known tendency to metastasize to bones, lymph nodes, and visceral organs.^1^^,^^2^ Standard treatments for advanced stages include hormone therapy to lower androgen levels and radiotherapy for localized tumors and metastases. While generally effective, metastasis during or after treatment remains a critical challenge, with 30 %–40 % of patients on hormone therapy and some post-radiotherapy patients developing progressive disease.^3^

Testicular metastasis from prostate cancer is exceptionally rare, accounting for less than 1 % of cases, largely due to anatomical and physiological barriers in the male reproductive system. Such metastases are often associated with poor outcomes, reflecting advanced systemic disease.^4^

This report discusses a 61-year-old male with isolated testicular metastasis after hormone therapy and radiotherapy, highlighting the importance of vigilant long-term monitoring for atypical metastatic patterns in prostate cancer patients.

## 2 Case presentation

The patient, a 61-year-old man, presented four years ago with a PSA level of 5.6 during a routine check-up. A prostate biopsy revealed Gleason score 9 across all cores. Digital rectal examination (DRE) indicated hardness in both prostate lobes, with no evidence of extracapsular extension. Imaging, including a CT scan of the abdomen and pelvis, showed no lymphadenopathy or signs of metastasis. A whole-body bone scan was also normal. Based on these findings, the patient was diagnosed with stage 2 prostate cancer (T2cN0M0) and deemed a candidate for androgen deprivation therapy (ADT) and radiotherapy by radiotherapist.

The patient received ADT for three years, following radiation therapy to the prostate (74 Gy/37 fractions). For the duration of treatment, his serum PSA levels were undetectable, and imaging revealed no lymph node enlargement, distant metastases, or skeletal involvement.

**Fig. 1:**
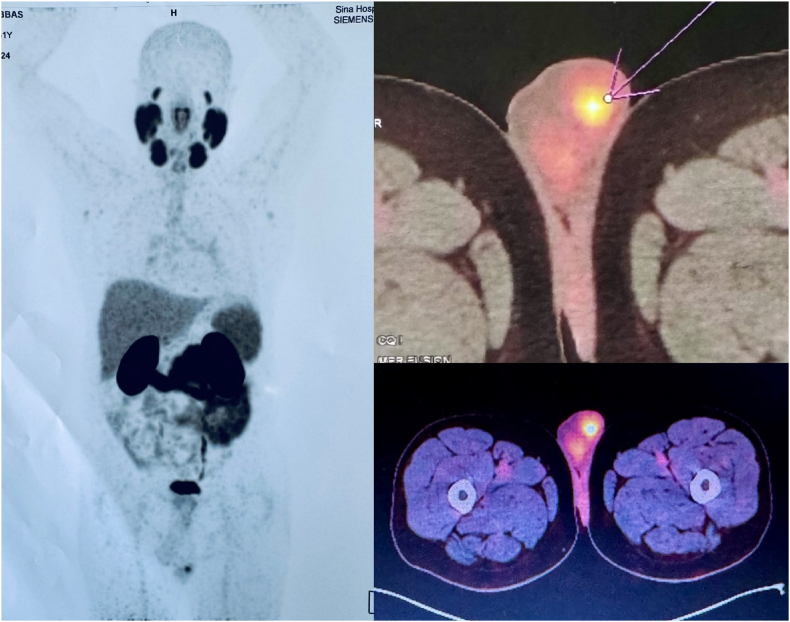
PET scan revealed focal increased Ga-PSMA uptake in the left scrotal region.

**Fig. 2:**
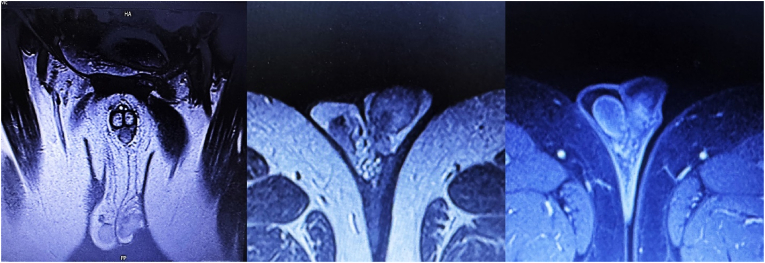
MRI revealed a tumoral nodule measuring 9 × 11 mm with enhancement in the left testicle.

**Fig. 3:**
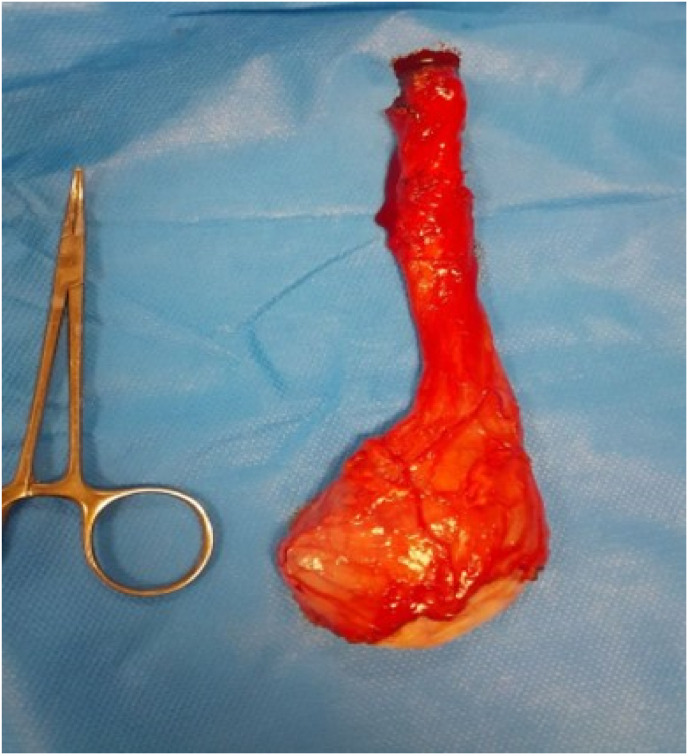
The specimen from left radical orchiectomy.

However, four months after completing ADT, the patient's PSA level rose to 0.21. A PSMA PET CT scan detected focal Ga-PSMA uptake in the left scrotal region (Fig. 1). Subsequent MRI revealed a 9 × 11 mm tumoral nodule with enhancement in the left testicle (Fig. 2). Serum markers, including alpha-fetoprotein, LDH, and BHCG, were within normal ranges. The patient underwent a radical orchiectomy (Fig. 3), and pathological analysis is discussed below: In microscopic examination, the section showed atrophic testicular tissue involved by a neoplasm composed of glands lined by monolayer of cells with conspicuous nucleoli. Tumor cells revealed positive immunoreactivity for CKAE1/AE3 and PSA and negative immunoreactivity for GATA3, CK7, Inhibin, calretinin, P63, CDX2, OCT3,4 which confirmed metastatic prostatic carcinoma.

We continued treatment with a GnRH agonist and Abiraterone following radical orchiectomy. In one-year follow up, the PSA levels is unmeasurable and the patient demonstrated favorable health outcomes with no evidence of local recurrence or distant metastases.

## 3 Discussion

Testicular metastases from prostate cancer (PCa) are exceedingly rare, with reported incidences ranging from 0.02 % to 2.5 %, excluding cases related to leukemia and lymphoma. Prostate cancer is the most common primary source of testicular metastases, accounting for approximately 15 % of such cases. Other sources include lung cancer, melanoma, skin cancers, colorectal cancer, and renal cell carcinoma.^5^ Many testicular metastases from PCa are identified post-mortem or during examinations of testes removed via therapeutic orchiectomy, emphasizing the difficulties in early detection.^6^

A significant distinction in our case report is that the patient was initially diagnosed at stage 2 (T2) and was not metastatic at the time of diagnosis. By contrast, kusaka et al. reported a patient at stage T3N1M1,^7^ while Bonetta et al. described an advanced case classified as T3B, eventually treated with radical prostatectomy.^4^

Our patient presented isolated metastasis to a single testicle, setting this case apart. Previous studies by M. Connelly et al., Rajesh Kamble et al., and Deb et al. documented metastases involving both testicles and other organs.^8^, ^9^, ^10^ Similarly, while Kamble et al. and Connelly et al. observed bilateral testicular metastases, our case was unique in that the metastasis was confined to one testicle. Furthermore, unlike other cases that often suggest poor prognosis with multiple metastases,^11^ our findings are more optimistic. Post-orchiectomy, the patient achieved unmeasurable PSA levels and demonstrated favorable health outcomes at the one-year follow-up. This underscores the importance of ongoing monitoring and the potential for positive results even in rare presentations of isolated testicular metastasis.

Mechanisms of PCa spread to the testis include retrograde venous extension, arterial or lymphatic embolism, and endocanalicular dissemination.^12^ Testicular metastasis risk is notably higher when the prostatic urethra is involved.^13^ In our case, retrograde venous extension likely contributed to the metastasis following radiation therapy.

Histologically, testicular metastases from PCa often resemble the primary prostatic adenocarcinoma.^13^ Patients with advanced PCa, particularly those with lymph node or skeletal involvement, frequently undergo prolonged androgen deprivation therapy (ADT).^14^ The interval from PCa diagnosis to the appearance of testicular metastases varies significantly, ranging from 2.5 to 15 years.^13^ This prolonged timeline raises questions about histological changes over time, with some studies suggesting a transition to different subtypes, such as small cell carcinoma.^15^ Tu et al. identified pathological features consistent with ductal or endometrioid adenocarcinoma, including tall columnar cells arranged in papillary, cribriform, and solid patterns. This indicates that metastatic tumors may develop as complex mixtures of tumor types derived from specific prostatic stem cells.^13^

This case highlights the rarity and clinical significance of testicular metastasis in PCa. Enhanced awareness, regular follow-up, and prompt intervention can improve patient outcomes in these challenging scenarios.

## 4 Conclusion

This case underscores the rarity of isolated testicular metastasis in prostate cancer, developing three years after hormone therapy and radiotherapy. It highlights the unpredictable nature of the disease and the need for vigilant PSA monitoring and imaging to detect late recurrences. The findings emphasize the importance of continued research into atypical metastatic patterns to enhance early detection and improve outcomes in oncology.

## CRediT authorship contribution statement

**Ali Tabibi:** Supervision, Project administration, Methodology, Investigation, Data curation, Conceptualization. **Mohammad Sajjad Zabihi:** Writing – original draft, Visualization, Methodology, Investigation, Data curation. **Mahyar Najarian:** Writing – original draft, Visualization, Methodology, Formal analysis, Data curation. **Mehdi Dadpour:** Writing – review & editing, Writing – original draft, Supervision, Methodology.
